# Fasciite nécrosante orbito-palpébrale, compliquant une pansinusite chez une patiente diabétique

**Published:** 2011-09-25

**Authors:** Idriss Benatiya Andaloussi, Meryem Abdellaoui, Salima Bhallil, Hicham Tahri

**Affiliations:** 1 Service d'ophtalmologie, CHU Hassan II, Fès, Maroc

**Keywords:** Fasciite nécrosante, Streptocoque pyogènes, paupières, orbite, diabéte

## Abstract

La fasciite nécrosante est un processus infectieux grave des tissus sous-cutanés avec une gangrène cutanée et des thromboses vasculaires. Elle reste grave mais heureusement rare. La localisation orbito-palpébrale est exceptionnelle. Le pronostic dépend de la précocité du diagnostic et du traitement. Nous rapportons un rare cas compliquant une pansinusite chez une patiente diabétique âgée de 17 ans.

## Introduction

La fasciite nécrosante est un processus infectieux grave des tissus sous-cutanés avec une gangrène cutanée et des thromboses vasculaires. Elle reste grave mais heureusement rare. La localisation orbito-palpébrale est exceptionnelle. Le pronostic dépend de la précocité du diagnostic et du traitement. Nous en rapportons un rare cas compliquant une pansinusite chez une patiente diabétique.

## Patient et observation

**Figure 1:**
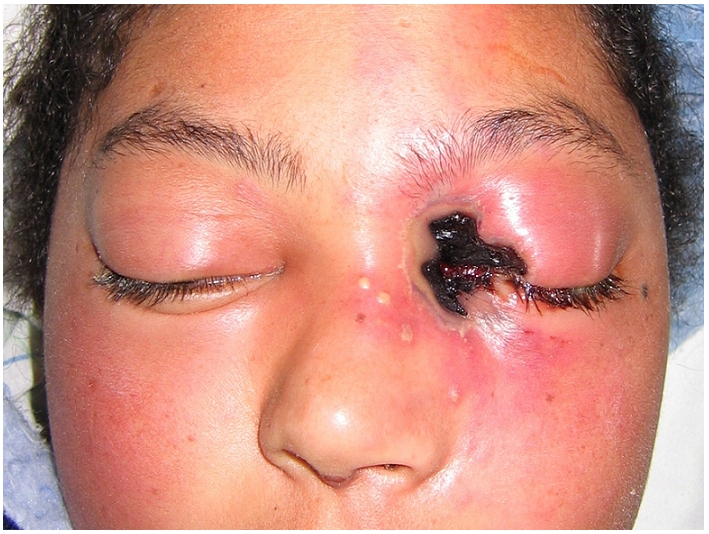
Nécrose cutanée de 2,3 cm de grand axe, localisée au niveau de la région canthale interne gauche, étendue au 1/3 interne des paupières supérieure et inférieure

**Figure 2:**
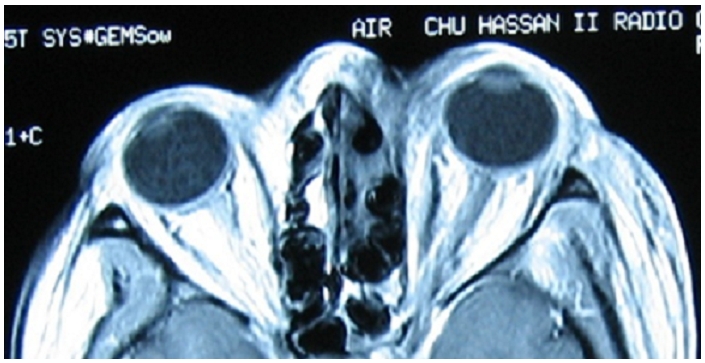
IRM orbito-céphalique montrant du côté gauche une nécrose superficielle des téguments de la région canthale interne

**Figure 3:**
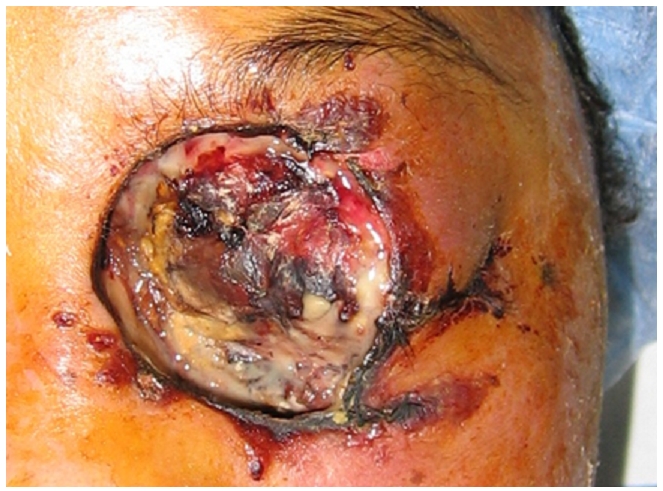
Extension de la zone de nécrose 48 heures après débridement des tissus nécrosés

Mlle F I, âgée de 17 ans, connue diabétique de type 2 mal équilibrée, présente une pansinusite évoluant depuis 15 jours mais non traitée. L’évolution est marquée l'installation d'un coma acidocétosique nécessitant son hospitalisation en ranimation pour équilibre. L'examen retrouve un œdème palpébral et chémosis, avec une nécrose cutanée à l'angle interne de l’œil gauche qui, selon la famille, évolue depuis plus d'une semaine. Cette nécrose cutanée, de 2,3 cm de grand axe, est localisée au niveau de la région canthale interne gauche et est étendue au 1/3 interne des paupières supérieure et inférieure (Figure 1). Le bilan biologique retrouve une hyperleucocytose à polynucléaires neutrophiles à 35.103 /mm3 et une CRP à 356 mg/dl. Après réalisation d'un prélèvement pour examen bactériologique, un traitement antibiotique à large spectre est alors démarré associant: vancomycine, métronidazole et rifampicine. Le prélèvement bactériologique objective un streptocoque beta hémolytique du groupe A sensible aux &beta; lactamines. L'IRM orbito-céphalique montre du côté gauche une nécrose superficielle des téguments de la région canthale interne, et une pansinusite (Figure 2). Un parage chirurgical des tissus gangrenés est alors réalisé au bloc opératoire et découvre une nécrose tissulaire arrivant au plan osseux. L’évolution est marquée, 48 heures après, par une aggravation de l’état neurologique de la patiente et une extension de la zone de nécrose (Figure 3).

**Figure 4:**
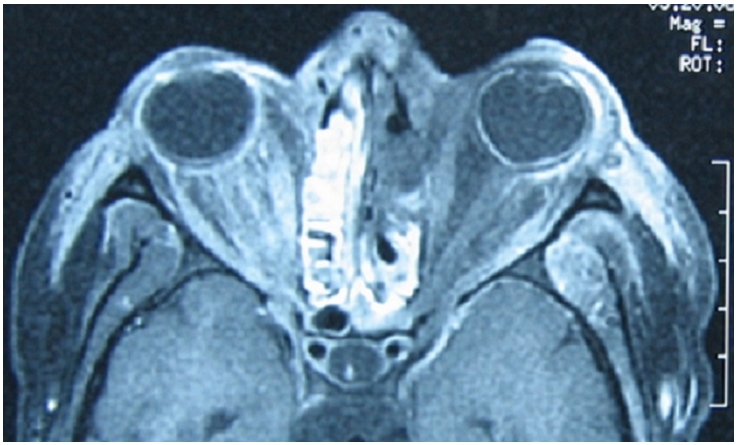
IRM orbitaire de contrôle montre une extension de la nécrose au sinus éthmoïdal et au contenu orbitaire

**Figure 5:**
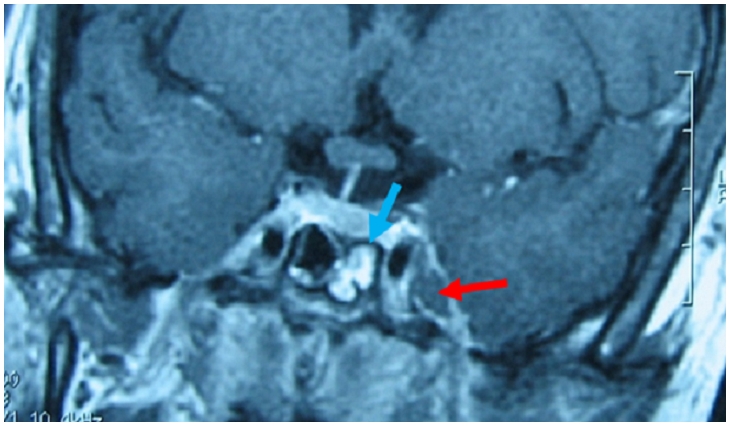
Thrombose du sinus caverneux gauche associé à une sphénoïdite à l'IRM cérébrale

L'examen ophtalmologique retrouve une semi-mydriase aréflexique gauche avec au fond d’œil montre une occlusion de l'artère centrale de la rétine. Une IRM orbito-céphalique de contrôle montre alors une extension de la nécrose au sinus éthmoïdal et au contenu orbitaire (Figure 4), et une thrombose du sinus caverneux gauche associé à une sphénoïdite (Figure 5).

**Figure 6:**
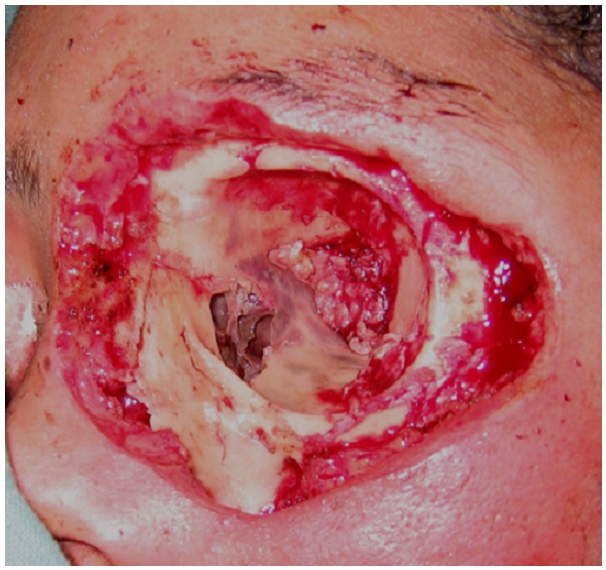
Aspect de nécrose osseuse de la paroi interne de l'orbite après exentération

Le geste chirurgical est alors complété par une exentération élargie aux paupières découvrant une nécrose osseuse de la paroi interne de l'orbite (Figure 6). La patiente est décédée 5 jours après dans un tableau de choc septique avec défaillance multi-viscérale.

## Discussion

La fasciite nécrosante est une pathologie infectieuse exceptionnelle à pronostic local et général sombre. Le processus touche essentiellement les extrémités, l'abdomen, le dos, les régions génitales et péri anales chez des patients de tout âge, sans prédilection de sexe ni de race [1–3]. La localisation orbito-palpébrale est rare: seuls 104 cas sont répertoriés de 1950 à 2010 dans une revue récente de la littérature [3]. Ceci est probablement dû à la richesse de la vascularisation de cette région permettant une meilleure diffusion des antibiotiques.

Généralement, la fasciite nécrosante est due à un Streptocoque β hémolytique du groupe A seul dans 50% des cas ou associée au Staphylocoque Aureus dans 18% [3]. Le diabète (notre cas), l'artério sclérose, l'alcoolisme, l'immunosuppression, et l'utilisation d'anti-inflammatoires non stéroïdiens sont les principaux facteurs prédisposant [3–5]. Dans près d'un tiers des cas aucune cause évidente n'est retrouvée [1,3]. Elle est souvent précédée d'un traumatisme même mineur [7], d'une chirurgie notamment après dacryorhinocystostomie [1,2,4] ou blépharoplastie [8], plus rarement d'une infection des voies aériennes supérieures comme chez notre patiente, d'une piqure d'insecte ou d'une extraction dentaire [4].

L'infection évolue très rapidement dans les 48 à 72 heures avec apparition d'un oedème inflammatoire puis d'une modification de la coloration cutanée qui devient bleue violacée. La douleur orbitaire, présente au début, disparait dès installation de la nécrose par destruction des filets nerveux [9]. Le marquage des bords de l′érythème tous les 1 à 2 heures peut être utile pour surveiller la progression de l'affection car elle se propage rapidement le long des plans aponévrotiques: la nécrose cutanée se développe au 4^e^-5^e^ jour avec suppuration sous-jacente entre le 8^e^ et le 10^e^ jour [1,3,4]. Des thromboses vasculaires avec ischémie chorio-rétinienne et cécité sont également rapportées [10]. Le diagnostic différentiel se fait essentiellement avec l’érysipèle, la gangrène gazeuse et le charbon palpébral. La tomodensitométrie ou l'imagerie par résonance magnétique permettent de mieux préciser l′étendue de l′infection et d′aider à planifier l'intervention chirurgicale. Cependant, il faut souligner que le diagnostic est clinique et que le traitement ne doit pas être retardé pour des raisons d′investigations biologiques ou radiologiques [9].

La prise en charge thérapeutique associe des mesures de réanimation et une antibiothérapie parentérale adaptées avec une large couverture (gram +, gram – et anaérobie) dans l'attente de l'isolement du germe [1,3].

Le débridement chirurgical des tissus nécrotiques doit être immédiatement associée afin de limiter l'extension du processus infectieux et de faciliter l'action de l'antibiothérapie intraveineuse [1,3,4]. Ce débridement devrait préserver le muscle sous-jacent et le bord des paupières pour simplifier une chirurgie reconstructive ultérieure qui peut être nécessaire pour prévenir les complications notamment l'ectropion et la kératite d′exposition [7]. Dans les cas limités aux paupières sans une importante morbidité associée, un traitement conservateur sans débridement avec auto-démarcation et couverture antibiotique sous surveillance rapprochée peut être réalisé [2,4]. L'utilité de l'oxygénothérapie hyperbare reste controversée 2. Des reconstructions palpébrales secondaires sont souvent nécessaires afin de réparer la destruction étendue des tissus cutanés et sous cutanés [1,6].

Malgré un traitement bien conduit, un choc toxique grave avec défaillance multiviscérale est susceptible de survenir pouvant entraîner le décès des patients dans 14,4% [3]. La fasciite nécrosante, touchant le cuir chevelu et la partie supérieure du la face, a un meilleur pronostic que les autres localisations, avec une mortalité de 12,5% [2]. Aucun cas de décès directement lié à la fasciite nécrosante limitée à la paupière n′a été signalé, bien que la morbidité soit importante. Le choc septique semble aussi être moins fréquent avec la fasciite nécrosante touchant uniquement les paupières, contrairement aux autres localisations notamment orbitaires.

Le pronostic est intimement lié à la précocité du la prise en charge thérapeutique, et à l'extension locale de l'infection [2,3]. Le décès de notre patiente est essentiellement dû au retard diagnostic et donc de prise en charge thérapeutique de plus d'une semaine.

## Conclusion

À travers notre observation, nous soulignons l'importance de la précocité du diagnostic et la rapidité de la prise en charge thérapeutique qui doit associer réanimation, antibiothérapie parentérale et parage chirurgical en urgence des zones nécrotiques.
